# AKT1/BRCA1 in the control of homologous recombination and genetic stability: the missing link between hereditary and sporadic breast cancers

**DOI:** 10.18632/oncotarget.203

**Published:** 2010-12-16

**Authors:** Josée Guirouilh-Barbat, Therese Wilhelm, Bernard S. Lopez

**Affiliations:** CNRS, UMR217, Institut de radiobiologie cellulaire et moléculaire, France

**Keywords:** AKT, BRCA1, DNA repair, homologous recombination

## Abstract

Endogenous replicative stress could be one trigger leading to tumor initiation: indeed, activation of the DNA damage response (DDR), considered the result of replicative stress, is observed in pre-cancerous cells; moreover, in hereditary breast cancers, almost all of the genes affected relate to the DDR. The most frequently mutated gene in hereditary breast cancers, BRCA1, is essential for homologous recombination (HR), a fundamental process for maintaining genome stability that permits the reactivation of blocked replication forks. Recent studies have established links between DDR and the oncogenic kinase AKT1, which is upregulated in about 50% of sporadic breast cancers. More specifically, the activation of AKT1 shows a deficient phenotype in BRCA1 and HR, revealing molecular similarities between hereditary and sporadic breast cancers. However, these results reveal a paradox regarding the physiological role of AKT1: in non-tumor cells, AKT1 promotes cellular proliferation, but consequently endangers genome integrity during replication if HR is inhibited. Since HR could itself lead to genetic instability, we propose that, under physiological conditions, moderate activation of AKT1 does not inhibit but prevents an excess of HR. The regulation of AKT1 would represent a fine transitory system for controlling HR and maintaining genomic integrity.

The coordination of a complex network of metabolic pathways ensures continued maintenance, duplication, and transmission of the genome. These metabolic pathways control the DNA damage response pathway (DDR) and bring together replication, recombination, DNA repair, chromosome segregation, and cell cycle control. However, in some common tightly-regulated processes, such as meiosis and the generation of the immune repertory, this network must allow/favor genetic diversity. Therefore, very precise regulation is necessary to control the equilibrium between genetic stability and diversity, while avoiding genetic instability. A defect in any of the actors in these pathways could result in genetic instability and a predisposition to tumor formation.

Certain types of cancers correspond to areas exposed to oncogenic agents (often genotoxic), such as UV radiation for skin cancer or tobacco for throat or lung cancer. However, we emphasize that many cancers develop without substantial exposure to exogenous carcinogens. Therefore, endogenous stresses must play crucial roles in the etiology of cancer. For example, mutating the BRCA1 or BRCA2 genes confers a hereditary predisposition to breast cancer in the absence of exposure to exogenous genotoxic agents.

## THE REPLICATION/RECOMBINATION INTERFACE, GENETIC INSTABILITY, AND CANCER

Among endogenous stresses, the spontaneous blocking of replication forks could constitute a risk for spontaneous tumor initiation. In fact, DNA replication forks are regularly blocked by a variety of endogenous stresses that can result from bulging regions in the DNA, regions of hybrid DNA/RNA, and from endogenous metabolism of the cell [1]. Furthermore, the prolonged arrest of these replication forks leads to the formation of double-strand breaks (DSBs) in the DNA, which can be taken care of by HR and non-homologous end-joining (NHEJ) [2-4]. It is therefore notable that the presence of DNA breaks and activation of the DDR pathway have been observed in the pre-cancerous stages of non-treated cells. This activation of the DDR pathway is considered the response to spontaneous replicative stress [5,6].

**Figure 1:**
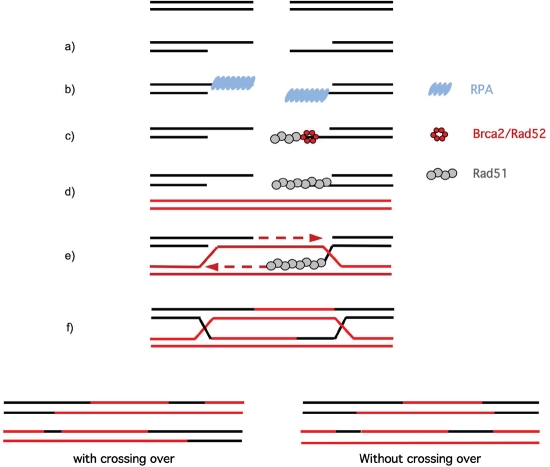
A Model for double-strand break repair by homologous recombination [78] a) A DSB in the DNA generates regions of ssDNA. This step is promoted by the RAD50/MRE11/NBS1 complex associated with CtIP in mammals [79-82]. b) ssDNA is covered by the RPA (Replication protein A) protein. c) RAD52 (in yeast) or BRCA2 (in mammals) displaces RPA from the ssDNA and loads the key protein for HR, RAD51. d) The ssDNA-RAD51 complex finds the intact homologous double-stranded DNA and promotes the exchange of identical strands and the hybridization of complementary strands. e) DNA polymerase fills in the gap and moves the displacement loop (D-loop). f) The cruciform junctions (Holliday junctions) are then formed. g) The resolution of the Holliday junctions depends on the direction of resolution and can proceed via the following two mechanisms: without crossing over or with crossing over (exchange of adjacent DNA sequences).

**Figure 2:**
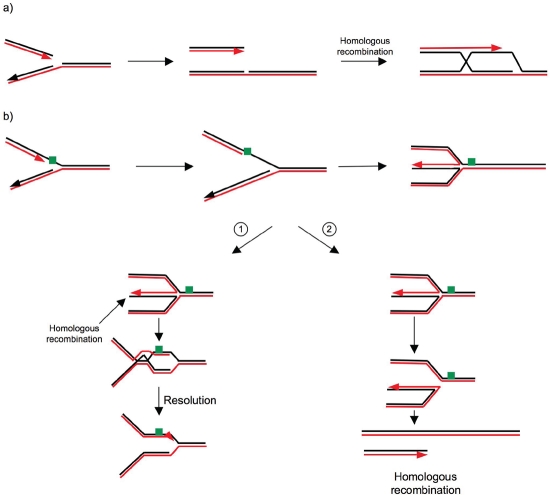
Examples of the role of HR in restarting blocked replication forks [14-16] a. A DSB can be generated by the collapse of a replication fork, for example due to a nick in the matrix. HR allows replication to restart by reinitiating it on the sister chromatid. b. When a fork reaches a blocking lesion, it can be reverted by generating a so-called “chickenfoot” structure. b-1. This structure has a DSB that can be used to initiate HR upstream of the blockage (given that the sequences are homologous). b-2. Alternatively, the cruciform structure can be resolved by specific endonucleases, which also generate DSBs. Replication can then be reactivated by HR using the sister chromatid.

One example illustrating this point of view is Bloom syndrome, which results in an increased predisposition to spontaneous tumor formation in all tissues, even those that are not directly exposed. Bloom syndrome is caused by inactivation of the BLM protein, a RecQ member of the helicase family, which plays an important role in resolving HR intermediates and controlling blocked replication forks [7-9]. Furthermore, among the 11 genes whose germline mutations are responsible for predispose to familial breast cancer, 10 are implicated in the DDR pathway [10,11]. Interestingly, the most frequently mutated genes, BRCA1 and BRCA2, play essential roles in HR [12,13], an essential process for maintaining genome integrity. The ubiquitous existence of this process in all living organisms highlights its biological importance. HR allows the repair of DSBs in DNA (Figure 1), as well as the reactivation of blocked replication forks (Figure 2) [14-16].

About one century ago, Theodor Boveri proposed that tumors arose from clonal development of a single aneuploid cell. He also proposed that this aneuploidy arose from unequal chomosome segregation, due to the presence of supernumerary centrosomes [17,18]. More recently, aneuploidy has been observed in precancerous lesions and tissues adjacent to tumors, suggesting a role in tumorigenesis [19-23]. This observation is therefore similar to activation of the DDR pathway and the detection of DSBs (see above). Centrosome duplication and genome replication should be coordinated to ensure faithful chrosomes segregation and exogenous stress that arrest replication generates supernumerary centrosomes [24-26]. It remains to be determined whether endogenous replicative stress and the presence of supernumerary centrosomes, which leads to aneuploidy, are connected at the molecular level and by what mechanisms. HR could be the link between these two processes. In fact, cells deficient in HR demonstrate both replicative stress, as characterized by a slowing of the replication speed [27] and an increased frequency of cells with supernumerary centrosomes [28-32]. It should be noted that these defects have been observed regardless of the HR gene affected, among them the tumor suppressor genes BRCA1 and BRCA2.

Finally, it is important to emphasize that the communication between replication and recombination could represent an effective target for anti-cancer therapeutics. In fact, PARP inhibitors generate replicative stress, leading to the formation of DSBs; tumors deficient in HR, such as those with defects in BRCA1 or BRCA2, would therefore be highly sensitive to these inhibitors [33,34].

## FAMILIAL VERSUS SPORADIC BREAST CANCERS: AKT1 AND THE DEREGULATION OF BRCA1

At present, the majority of mutations that confer a predisposition to familial breast cancer affect genes implicated in DDR, specifically at the interface of replication and recombination. The most frequently mutated gene, BRCA1, plays an important role in HR [12,35]. This overrepresentation of genes implicated in a particular pathway highlights the importance of the DDR pathway and the communication between replication and recombination in the etiology of breast cancer. This begs the question of whether the etiology of sporadic breast cancers shares the same mechanisms as those of hereditary breast cancers. This question is perhaps more relevant because sporadic cases of breast cancer are far more common than hereditary cases; in fact, hereditary breast cancer are consider to represent 5 to 10 %, and mutation of BRCA1 1 to 2%, of all cases [36,37].

The extremely diverse characteristics of tumors grouped in a common category (for example, the set of spontaneous breast cancers) represent an important obstacle to understanding the underlying molecular mechanisms in an unifying view. However, it is noteworthy that, in a number of studies, the oncogenic kinase AKT1 has been shown to be upregulated in 40-60% of sporadic breast cancers and 40% of sporadic ovarian cancers [38-40]. Furthermore, AKT1 can phosphorylate BRCA1 *in vitro* [41]. Therefore, it is important to determine whether the activation of AKT1 in sporadic cancers leads to a phenotype similar to that observed in familial cancers. AKT1 is involved in the PI3 Kinase/PTEN/AKT1 signaling pathway and responds to extracellular stimuli, including growth factors and hormones [42]. PTEN is a tumor suppressor gene and an antagonistic inhibitor of AKT1 (inactivation of PTEN leads to the activation of AKT1). It is noteworthy that i) PTEN is one of the mutated genes in familial breast cancers [43,44], and decreased amount of the PTEN protein is observed in 25% of breast cancers [45]; ii) mutations in PTEN are associated with Cowden syndrome, in which the probability of developing breast cancer reaches 30% [46]; iii) the inactivation of PTEN leads to an increase in genetic instability [47-50]; iv) cells lacking PTEN show elevated levels of spontaneous DSBs [49,50] and decreased expression of the recombinase RAD51 [50-52], which lead to the defective repair of DSBs by HR; and v) tumors or cells lacking PTEN are sensitive to PARP inhibitors [52,53].

Rencently, several studies have been demonstrating a relationship between AKT1 and DDR. For instance, AKT1 destabilizes p53 via the phosphorylation of Mdm2 [54]; AKT1 controls the basal expression of XRCC1 [55]; the activation of AKT1 leads to the sequestration of CHK1 to the cytoplasm [47-49], and AKT1 phosphorylates and prevents the activation of CHK1 by ATR/ATM [56]; finally, AKT1 reduces the abundance of γ-gH2AX foci [57,58] in asynchronous cells, and inhibits the activation of CHK1 and the repair of DSBs at the end of G2 [59].

More directly related to the questions discussed here, the overexpression of AKT1 has been shown to promote the sequestration of BRCA1 and RAD51 to the cytoplasm [38,60]. This sequestration of BRCA1 and RAD51 to the cytoplasm has been observed both in cultured cell lines and in 60% of sporadic breast cancer tumors, in which it is correlated with the level of AKT1 activation [38]. Delocalizing BRCA1 and RAD51 to the cytoplasm inhibits the nuclear functions of BRCA1, such as the recruitment to sites of damage after exposure to ionizing radiation and the control of HR. Consistent with the phenotype of cells that are mutated in components of HR, cells overexpressing AKT1 have a higher frequency of supernumerary centrosomes [60]. Furthermore, the AKT1 signaling pathway negatively regulates the expression of BRCA1 mRNA [61]. Therefore, the over-activation of AKT1, which occurs in about half of all sporadic breast cancers, leads to a phenotype similar to that of *brca1*^−/−^ cells, without the need for mutating the BRCA1 gene. Moreover, it seems, that a lack of BRCA1 is linked to a constitutive activation of the AKT1 signaling pathway. Because BRCA1 is the most frequently mutated gene in hereditary breast cancers, the relationship between AKT1 and BRCA1 could constitute the missing molecular link between sporadic and familial breast cancers.

Conversely, BRCA1 negatively regulates AKT1 by inducing its degradation [61]. In addition, a lack of BRCA1 activates the AKT1 pathway by causing disappearance of the PTEN protein, which is observed in 82% of hereditary breast cancers linked to BRCA1 [62].

Epidemiological studies have concluded that hormone substitution leads to an increased risk for breast cancer [63-65]. Because AKT1 activity is inducible by hormones [66,67], it is tempting to speculate that, for certain people, hormonal treatments could lead to high and chronic activation of AKT1, thus altering the functions of BRCA1, and therefore to predispose to breast cancer.

The negative impact of AKT1 on HR and on BRCA1 localization, resulting in a BRCA1-deficient phenotype (without requiring mutation in the BRCA1 gene) suggests that tumors with hyperactivated AKT1 might be sensitive to PARP inhibitors. In agreement with this model, PTEN deficient tumors are hypersensitive to PARP inhibitors [52]. Therefore the high frequency of AKT1 activation in sporadic breast cancer opens promizing new avenues for therapy. However, because AKT1 activation also protects against cell death and because of the highly pleiotropic regulation of AKT1, the molecular characterization of AKT1 impact on HR becomes an essential issue.

## THE PARADOX OF AKT1: A RHEOSTAT OF HR?

The inhibition of HR by AKT1 is mechanistically consistent with its role in breast cancer, i.e., under pathological conditions. However, under physiological conditions, this reveals a paradox, notably in response to growth factors, because AKT1 plays a role in cellular proliferation (for a review, see [68]). A number of studies have demonstrated that the PI3K pathway is involved in the G1 to S phase transition via i) the inactivation of GSK3β, and the stabilization of cyclin D and c-myc, ii) inhibition of the Forkhead family of transcription factors leading to a decrease in p27^Cip1^, and iii) inactivation of p21 and p27 via direct phosphorylation by AKT1 [68]. Because cellular proliferation requires genome replication, the inhibition of HR by the activation of AKT1 thus presents a risk to maintaining genome stability. Two possible solutions can resolve this paradox:

### 1- Level and duration of AKT1 activation

We must point out that the level and duration of AKT1 activation are very different between physiological and pathological conditions. The activation of AKT1 is moderate and mostly transient under physiological conditions, but is stronger and constant (generally due to upstream deregulation, e.g., the inactivation of PTEN) under pathological conditions, thus accounting for the pathological phenotype. For example, the activation of AKT1 by a growth factor (heregulin β1) or by hormones (IGF-1 and estrogen) (in other words, physiological activation mechanisms) promotes the nuclear localization of BRCA1 [69,70]. Moreover it is also reasonable to suggest that the consequences of AKT1 activation may vary based on cell type.

### 2- Risk of excess HR initiation on genetic stability

If HR is an essential process for maintaining genome integrity, it is also a double-edged sword, because it can also generate genetic instability:

A - On one hand, defects in HR lead to genetic instability and increased mutagenesis.

**Figure 3:**
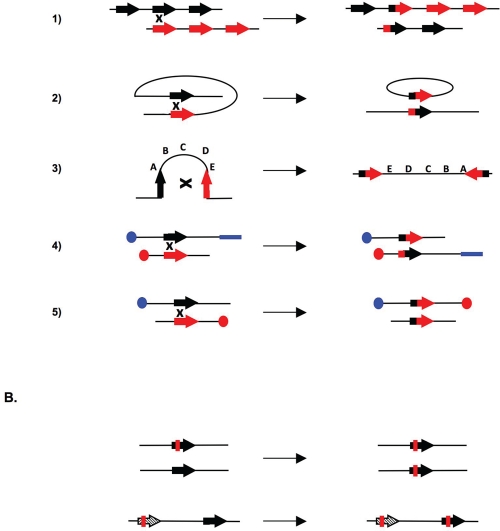
Genetic instability linked to excess of homologous recombination A. Chromosomal rearrangements resulting from crossing over (CO). 1. CO between repetitive sequences on two chromosomes or during an unequal sister chromatids exchange, resulting in an amplification on one molecule and a deletion on the other. 2. Intra-chromatid CO between two direct repeats, resulting in excision of the internal fragment. 3. Intra-chromatid CO between inversely oriented sequences, resulting in inversion of the internal fragment. 4. and 5. Inter-chromosomal CO. Depending on the orientation of the sequence with respect to the centromere (blue or red circles), the process will generate a translocation (4) or a dicentric chromosome and an acentric chromosome (5). B. Genetic modifications resulting from gene conversion without crossing over. Top: between two heteroalleles, leading to a loss of heterozygosity. Bottom: gene conversion between a pseudogene (hatch-marked), which often contains stop codons, and a gene, resulting in inactivation of the gene. Mutations are shown in red.

On the other hand, the genome contains many repetitive sequences, and HR between these sequences can lead to chromosomal rearrangements and therefore genetic instability (Figure 3). Severe chromosomal rearrangements, such as deletions, inversions, duplications, and translocations requiring repetitive sequences have been observed in various human pathologies [71-74].

B - Unresolved HR intermediates are toxic and can generate genetic instability [75].

Therefore, maintaining genome stability benefits from the avoidance of excess HR initiation. This point is especially crucial during the S phase, because HR occurs preferentially during the S phase [76,77]. In addition, the substrate for HR is single-stranded DNA (ssDNA) covered with the RPA protein (Figure 1). Because RPA is a protein implicated in replication, RPA complexed to ssDNA is present throughout the genome during replication. If HR were initiated every time that RPA bound to ssDNA, it would lead to complete disruption of the genome. Therefore, it is necessary to precisely control recombination to maintain genome stability and avoid genetic instability, particularly during S phase. Signaling downstream of the ssDNA-RPA complex might be different in the replication *vs.* the HR intermediates, following the resection step. Moreover, helicases have been shown participate to the maintenance of genome stability by disrupting abortive HR intermediate [75]. However, it is necessary to precisely control recombination at multiple levels to maintain genome stability, particularly during S phase. We propose that AKT1 is an upstream regulator by preventing the excess of HR initiation.

### 3- A potential role for AKT1 under physiological conditions

The previous explanations suggest that in non-stressed cells, the transient and moderate activation of AKT1 avoids excess HR, which could be harmful to genome stability, without completely repressing it. The activation of AKT1 by extracellular factors (growth factors, hormones, interleukins), which depends on the combination and concentration of these extracellular factors, and of the cellular receptors of these factors, should therefore allow for a subtle and transitory regulation of HR. Under physiological conditions, AKT1 would play a role as a rheostat to precisely regulate HR. For example, in human fibroblasts, the growth factor FGF (fibroblast growth factor) activates AKT1 and represses excess HR without inhibition of HR; in fact, the level of HR never decreases by more than 50% of the level in non-stimulated cells [38]. In contrast, the strong and constant activation of AKT1 must strongly affect the subtle regulation of HR, leading to an important disequilibrium and the complete inhibition of HR, thus promoting an abnormal or pathological condition.

The characterization of the molecular mechanisms that allow AKT1 to modulate HR, and more generally DNA repair, represents an important focus of future research. Uncovering these processes will allow better understanding of the mechanisms that maintain genetic stability and result in spontaneous tumor development and to optimize cancer therapy.
